# Effect of simulated acidification on soil properties and plant nutrient uptake of eggplant in greenhouse

**DOI:** 10.3389/fpls.2025.1558458

**Published:** 2025-03-31

**Authors:** Tianqi Wang, Leixin Yu, Zhen Wang, Chuang Yang, Feiyu Dong, Diwen Yang, Haijun Xi, Zhouping Sun, Roland Bol, Muhammad Awais, Lijuan Yang, Hongdan Fu

**Affiliations:** ^1^ College of Land and Environment, Shenyang Agricultural University, Shenyang, China; ^2^ College of Horticulture, Shenyang Agricultural University, Shenyang, China; ^3^ Shenyang Urban Construction University, Shenyang, China; ^4^ College of Agricultural Science and Technology, Shandong Agriculture and Engineering University, Shandong, Jinan, China; ^5^ Shenyang Hengxin Technology Management Consulting Service Co., Ltd, Shenyang, China; ^6^ Chaoyang Agricultural Development Service Center, Chaoyang, China; ^7^ Institute of Bio- and Geosciences, IBG3 Agrosphere, Forschungszentrum Juelich, Juelich, Germany

**Keywords:** greenhouse, eggplant, soil pH, elements nutrient, macro elements, trace elements

## Abstract

Soil acidification adversely affects plant growth and development by decreasing the accessibility of roots to essential nutrients. Thus, it decreases crop yield. However, there has been a lack of systematic research on how soil acidification influences nutrient absorption in eggplant cultivated in greenhouse. To address this research gap, an experiment was conducted in a greenhouse with seven different acidity levels (4.5, 5.0, 5.5, 6.0, 6.5, 7.0, 7.5), achieved by adding dilute H_2_SO_4_. The findings indicated that the soil organic matter (SOM) content at pH 4.5 decreased by 49% - 50% compared to pH levels of 7.0 - 7.5. In addition, the levels of exchangeable aluminum (Al^3+^) and soil electrical conductivity (EC) were highest at pH 4.5, with increases of 82 -88 mg kg^-1^ and 1.78 - 1.82 ms cm^-1^, respectively, compared to pH 7.0 - 7.5. The total nitrogen (TN), phosphorus (TP), and potassium (TK) content in the soil declined as acidity increased, reaching their lowest levels of 0.59, 0.42, and 3.79 g kg^-1^ at pH 4.5. Among the available nutrients, only potassium levels did not exhibit significant variation across treatments. However, the levels of macro elements in the soil consistently decreased, while the concentrations of trace elements (Fe, Cu, Zn) increased with rising acidity; conversely, the levels of other trace elements (B, Mo, Mn) decreased. The amounts of exchangeable calcium (Ca^2+^) and magnesium (Mg^2+^) at pH levels of 4.5 - 5.0 dropped by 61% - 66% and 70% - 78%, respectively, compared to pH 7.5. Further analyses indicated that soil pH values between 6.0 - 4.5 reduced the nutrient absorption capacity of eggplant, with the lowest nutrient content observed at pH 4.5. Mantel analyses confirmed that soil pH significantly affects plant nutrient uptake. This research provides both theoretical insights and practical guidance for the effective management of vegetable soil in greenhouse.

## 1 Introduction

The vegetables industry in China has experienced rapid development since the 20^th^ century. According to the 2019 census conducted by the National Bureau of Statistics of China (2020), the area dedicated to vegetables cultivation in the country has reached 20 million hectares (Shen et al., 2021). This cultivated area has been gradually increasing and ranks first globally (Puissant et al., 2019; Lv et al., 2020; Yuan et al., 2024). The advent of greenhouse cultivation has become a vital component of agricultural practices, establishing a stable ecological environment conducive to vegetables growth. It helps to extend the planting season, increase yields, boost farmers’ incomes, and ensure a year-round supply of vegetables (Bai et al., 2020; Das et al., 2022). Despite the numerous advantages offered by facility agriculture, it simultaneously confronts a succession of challenges (Zhou et al., 2021).

The widespread adoption of facility agriculture and the practice of multi-stubble cultivation in numerous greenhouse have contributed to an increased prevalence of soil acidification. For example, the overuse of ammonium-based nitrogen fertilizers has been shown to facilitate the accumulation of hydrogen ions (H^+^) in the soil, thereby elevating soil acidity (Li et al., 2018; Zhang et al., 2022b; Wang et al., 2023a, b). In addition, the application of various acidic fertilizers, including potassium chloride, ammonium sulfate, and ammonium chloride, in facility production has resulted in the retention of significant quantities of acidic ions such as sulfate (SO_4_^2-^), chloride (Cl^-^), and nitrate (NO_3_^-^) in the soil over extended cultivation periods. These ions are either not absorbed or are infrequently utilized by plant, leading to their interaction with H^+^ in the soil and a subsequent reduction in soil pH (Benelli et al., 2020; Liang et al., 2024). Research conducted by Lv et al. (2020) indicates that the long-term application of nitrogen fertilizers contributes to decline in soil pH. This includes the use of nitrogen-containing alkaline fertilizers (such as ammonium bicarbonate and ammonia) or neutral fertilizers (such as ammonium nitrate and urea), where excessive ammonium (NH_4_^+^) is converted into nitrate (NO_3_^-^) and nitrite (NO_2_^-^), further exacerbating soil acidification. Notably, ammonium nitrogen also promotes the production of substantial amounts of H^+^, which perpetuates the increase in soil acidity (Hafez et al., 2020; Lv et al., 2020; Zhang et al., 2024).

Soil nutrients play a crucial role in agriculture and plant growth (Yang et al., 2024). A significant amount of soil exchange calcium (Ca^2+^) has been leached out due to soil acidification, leading to severe damage to soil structure and compaction (Babin et al., 2019; Liang et al., 2024). The inadequate development of soil aggregates impairs the soil’s capacity to act as a buffer and facilitate aeration (Zhang et al., 2016; Latifah et al., 2017; Arwenyo et al., 2023). As soil acidity changes, the availability of phosphorus (P), potassium (K), calcium (Ca), magnesium (Mg), molybdenum (Mo) and other elements also reacts accordingly (Shi et al., 2018; Yao et al., 2024). Soil acidification makes phosphorus and potassium easily fixed, low uptake and utilization of medium and trace elements, and weak root growth, resulting in increased fertilizer use but poor plant growth (Wang et al., 2020b; Zhang et al., 2022a).Soil acidity not only leads to deficiencies in essential nutrients but also contributes to the accumulation of harmful substances, such as exchangeable aluminum (Al^3+^) in the soil (Guo et al., 2018; Munyaneza et al., 2024). When the soil pH drops below 5.5, Al^3+^ becomes highly soluble in the soil solution, significantly affecting the fluidity of phospholipids within root cell membranes and potentially leading to membrane peroxidation. This phenomenon can subsequently result in stunted growth of lateral roots and root tips (Ballagh et al., 2023). In addition, elevated concentrations of NH_4_^+^, produced through microbial nitrification, can inhibit plant growth (Shoghi Kalkhoran et al., 2021). Such as conditions may cause leaves to lose their green pigmentation, disrupt biochemical processes, alter energy transfer mechanisms, and ultimately impede overall plant development (Fan et al., 2024).

Eggplant (*Solanum melongena L.*) is one of the most significant greenhouse vegetables crop in China, contributing approximately 64.48% of the global output. Due to its high economic value and market demand, the excessive use of nitrogen fertilizers in greenhouse conditions has become a prevalent issue. The increased fertilizer application can lead to soil acidification and other problems, which seriously threaten the eggplant market and industry (Wang et al., 2020a; Adamczewska-Sowińska et al., 2022).

Despite the significance of soil acidification, there is a lack of research examining its effects on the growth and development of eggplant, as well as its influence on nutrient distribution within the soil and plant tissues. Consequently, we designed a pot experiment to simulate a soil acidification environment by applying dilute sulfuric acid under controlled greenhouse conditions. This approach is based on the understanding that soil acidification primarily results from an increase H^+^. Dilute sulfuric acid, which can fully dissociate in aqueous solutions, serves as a significant source of H^+^ ions for the soil. Unlike alternative acidification methods, eggplant was chosen as the experimental subject for this study. The primary goals of our current research were: 1) to study the effects of soil acidification on soil properties, and 2) to explore the effects of soil acidification on nutrient absorption in eggplant.

## 2 Materials and methods

### 2.1 Experimental site and design

The experiment was conducted in greenhouse (41°48’N, 123°25’E) at Shenyang Agricultural University, Liaoning Province, China. The eggplant variety “Xi ‘an green” was used as a test material. We initially collecteded soil from an adjacent field to the greenhouse, the soil type is meadow soil, the basic properties of the original soil are detailed in **Supplementary Table S1**. Polyethylene plastic pots, each measuring 27.00 cm in width and 30.00 cm in height, were utilized to contain equal. Before planting, 190.00g of chicken manure was applied to each pot as a base fertilizer, and 15.00g of NPK (15:15:15) compound fertilizer was applied to each pot after planting. We established seven acidity levels and repeated each treatment four times. The pH of each treatment was checked every seven days and H_2_SO_4_ (with corresponding acid solution concentrations of 0.10, 0.13, 0.20, 0.53, 0.70, 0.95 and 1.00 mL L^-1^) was added as needed to maintain the target soil pH values (**Supplementary Table S2**).

### 2.2 Sample collection

On 28^th^ June 2023, plants were carefully uprooted, and the fresh weights of the stem, root, and leave were recorded before being transferred to an oven for drying. Subsequently, the dry weights of the plant were measured and used to determine nutrient absorption in the dried plant parts. Meanwhile, the soil auger was used to collect soil samples from a depth of 0 - 10.00 cm at five different points in each pot for each treatment. The fresh soil was passed through 2.00 mm sieve to remove stones, pebbles and roots. The collected samples were left to dry at room temperature for the measurement of chemical properties.

### 2.3 Measurement methods

#### 2.3.1 Determination of soil basic properties

Soil pH (deionized water: soil, 2.5: 1) was measured using a standard pH meter (pHS - 25). Soil electrical conductivity (EC) (deionized water: soil, 5: 1) was determined by a conductometer (DDS - 241 307A, INESA Scientific Instrument, Shanghai, China). Total nitrogen (TN) was measured through an automatic Kjeldahl distillation titration method (Cabrera and Beare, 1993). Total calcium (TCa), total magnesium (TMg), total copper (TCu), total iron (TFe), total manganese (TMn), total zinc (TZn) and total potassium (TK) in soil were determined by H_2_SO_4_ - HClO_4_ digestion method and analyzed by flame spectrophotometer. Total phosphorus (TP) was determined by H_2_SO_4_ - HClO_4_ digestion method and analyzed by ultraviolet spectrophotometer (Nobile et al., 2020). Bao (2000) method was utilized to measure Alkali-hydrolyzed nitrogen (AN). The NaHCO_3_ leaching molybdenum antimony colorimetric technique was used to measure available phosphorus (AP). Leaching NH_4_OAC (pH=7.00) solution in 1.00 mol L^-1^ was used, and then the rapid available potassium (AK), exchange calcium (Ca^2+^) and exchange magnesium (Mg^2+^) were analyzed by flame photometer (Bao, 2000). Soil organic matter (SOM) was determined by H_2_SO_4_ - K_2_Cr_2_O_7_ titration (Kalembasa and Jenkinson, 2006). After digestion with HNO_3_ - HClO_4_, total molybdenum (TMo) was determined by ICP - MS method, and total boron (TB) was measured by methimine colorimetry. The available molybdenum (AMo) was determined by ICP - MS method after leaching with oxalic acid and ammonium oxalate. Soil available copper (ACu) and available zinc (AZn) were analyzed by 0.10mol L^-1^ HCl extraction and flame spectrophotometer. The leaching was carried out by DTPA - TEA with flame separation Soil available manganese (AMn) and available iron (AFe) were analyzed by optical photometer. Leaching with boiling water and determination of available boron (AB) by methylimine colorimetry (Bao, 2000).

#### 2.3.2 Plant nutrient determination

Plant total potassium (PK), calcium (PCa), magnesium (PMg), copper (PCu), iron (PFe), zinc (PZn) and manganese (PMn) were analyzed by flame spectrophotometer after digestion with H_2_SO_4_ - H_2_O_2_, and plant total nitrogen (PN) was measured through an automatic Kjeldahl distillation titration method. Total phosphorus (PP) was determined by vanadium-molybdenum yellow colorimetric method. Total boron (PB) was analyzed by curcumin colorimetric method (Bao, 2000).

### 2.4 Statistical analyses

Excel 2021 was utilized for basic data collation and processing analyses, SPSS Statistics 26.0 (IBM, New York, USA) was employed to conduct one-way ANOVA (*p*<0.05), while Duncan’s test was used for multiple comparisons of the data. RDA and Pearson correlation analyses were performed to investigate the relationships between soil chemical properties and soil nutrients. In addition, Principal Coordinate Analyses (PCA) was conducted using the vegan package in R software to compare nutrient absorption differences among various treatments. Mantel correlation was applied to analyses the relationship between soil chemical properties and plant nutrients. The Origin2021 was used to visualize the results.

## 3 Results

### 3.1 Effect of simulated acidification on soil basic chemical properties and nutrients of eggplant in greenhouse

#### 3.1.1 Effect of acidification on soil EC, SOM and Al^3+^

The results showed that soil organic matter (SOM) content decreased by 49% - 50% to 5.57 g kg^-1^ at pH 4.5 compared with pH 7.0 - 7.5 (**Table 1**). Meanwhile, the soil electrical conductivity (EC) value at pH 4.5 was significantly different from that at pH 7.5, and the exchangeable aluminum (Al^3+^) content in soil reached the maximum value (113 mg kg^-1^) at pH 4.5, which was 4.42 times that at pH 7.5. Soil Al^3+^ content at pH 7.0 and 7.5 was significantly different from that at pH 4.5 and 5.0 (**Table 1**).

**Table 1:** Effects of acidification on EC, SOM and Al^3+^.

Treatment pH	EC(ms cm^-1^)	SOM(g kg^-1^)	Al^3+^(mg kg^-1^)
7.50	0.19 ± 0.01e	11.08 ± 0.45a	25.65 ± 4.48d
7.00	0.23 ± 0.01e	10.90 ± 0.95a	31.72 ± 2.02d
6.50	0.56 ± 0.02d	9.19 ± 0.99ab	50.62 ± 6.46c
6.00	0.68 ± 0.01d	9.53 ± 0.33ab	62.10 ± 3.31c
5.50	1.22 ± 0.08c	8.51 ± 0.33ab	93.82 ± 2.78b
5.00	1.70 ± 0.03b	7.98 ± 0.49b	99.90 ± 7.31ab
4.50	2.01 ± 1.07a	5.57 ± 1.42c	113.40 ± 11.76a

The same letter in the same column indicates that the results are not significant (P<0.05).

#### 3.1.2 Effect of acidification on macro elements content

Soil total nitrogen (TN) peaked at pH 7.5, 55% higher than at pH 4.5. Alkali-hydrolyzed nitrogen (AN) increased with acidity, ranging from 51 - 81 mg kg^-1^ (**Table 2**). The content of soil total phosphorus (TP) at soil pH7.5 was significantly higher than that at soil pH 4.5. Available phosphorus (AP) varied greatly, with the highest (278 mg kg^-1^) at pH 4.5 and the lowest (134 mg kg^-1^) at pH 6.5, and the difference between treatments was significant (**Table 2**). The data indicated that the trend in the variation of total potassium (TK) and available potassium (AK) content in the soil decreased as soil pH decreased, the soil pH content at 7.5 was significantly different from that at 4.5 (**Table 2**).

**Table 2:** Effect of acidification on soil nutrients of eggplant in greenhouse.

pH	7.5	7.0	6.5	6.0	5.5	5.0	4.5
TN(g kg^-1^)	1.30 ± 0.06a	1.00 ± 0.21ab	0.87 ± 0.08bc	0.81 ± 0.04bc	0.72 ± 0.08bc	0.75 ± 0.08bc	0.59 ± 0.13b
TP(g kg^-1^)	0.66 ± 0.05a	0.60 ± 0.02ab	0.51 ± 0.13ab	0.50 ± 0.04ab	0.50 ± 0.03ab	0.48 ± 0.01ab	0.42 ± 0.03b
TK(g kg^-1^)	6.12 ± 1.10a	5.18 ± 0.15ab	4.94 ± 0.34ab	4.90 ± 0.09ab	4.39 ± 0.63ab	4.52 ± 0.71ab	3.79 ± 0.60b
TCa(g kg^-1^)	2.81 ± 0.08a	2.44 ± 0.12a	1.97 ± 0.14b	1.77 ± 0.20b	1.57 ± 0.14b	0.93 ± 0.23c	0.63 ± 0.05c
TMg(g kg^-1^)	8.73 ± 0.36a	7.32 ± 0.87ab	6.11 ± 1.01abc	7.12 ± 1.09ab	5.84 ± 1.46bcd	3.58 ± 0.36cd	3.20 ± 0.23d
TFe(g kg^-1^)	9.13 ± 0.48c	9.09 ± 0.22c	10.98 ± 0.35b	11.00 ± 0.30b	11.00 ± 0.35b	12.27 ± 0.44ab	13.71 ± 1.22a
TMn(mg kg^-1^)	189 ± 25a	208 ± 33a	185 ± 27a	156 ± 13a	167 ± 10a	194 ± 16a	173 ± 23a
TCu(mg kg^-1^)	6.09 ± 0.59b	6.06 ± 0.38b	7.32 ± 0.74b	6.46 ± 0.38b	7.26 ± 0.81b	7.63 ± 0.63b	11.29 ± 0.44a
TZn(mg kg^-1^)	23.17 ± 0.83c	26.02 ± 0.53bc	28.92 ± 1.15ab	29.61 ± 2.56ab	30.03 ± 0.21ab	31.75 ± 1.86a	33.44 ± 2.27a
TB(mg kg^-1^)	460 ± 14a	429 ± 20ab	368 ± 30bc	311 ± 21cd	280 ± 31de	235 ± 12ef	215 ± 9.3f
TMo(mg kg^-1^)	8.37 ± 0.96a	8.22 ± 1.04a	6.85 ± 0.16a	4.35 ± 0.85b	2.63 ± 0.47bc	1.99 ± 0.46c	1.58 ± 0.30c
AN(mg kg^-1^)	51 ± 6.47c	54 ± 2.26c	54 ± 2.26c	59 ± 2.99bc	67 ± 3.20b	67 ± 3.78b	817 ± 3.61a
AP(mg kg^-1^)	140 ± 15.20b	156 ± 7.18b	134 ± 9.64b	176 ± 13b	178 ± 17b	178 ± 7.40b	278 ± 20.19a
AK(mg kg^-1^)	870 ± 2.35a	810 ± 31ab	784 ± 28bc	749 ± 9.51bc	731 ± 25c	669 ± 24d	640 ± 2.72d
Ca^2+^(mg kg^-1^)	1227 ± 54a	1053 ± 4.79b	1052 ± 22b	827 ± 94c	756 ± 36c	473 ± 42d	414 ± 40d
Mg^2+^(mg kg^-1^)	147 ± 8.51a	132 ± 11.82a	114 ± 7.95ab	94 ± 23.62b	79 ± 8.24bc	45 ± 9.77cd	33 ± 8.41d
AFe(mg kg^-1^)	9.09 ± 0.61d	10.85 ± 0.29d	19.50 ± 5.34d	38.46 ± 3.34c	51.30 ± 6.04c	68.00 ± 6.21b	90.36 ± 4.52a
AMn(mg kg^-1^)	4.89 ± 0.39c	4.14 ± 0.30c	7.06 ± 1.11c	12.22 ± 0.49b	15.89 ± 1.18ab	18.85 ± 3.42a	20.19 ± 1.85a
ACu(mg kg^-1^)	0.20 ± 0.02d	0.28 ± 0.02c	0.31 ± 0.01bc	0.30 ± 0.02bc	0.34 ± 0.00ab	0.34 ± 0.01ab	0.37 ± 0.01a
AZn(mg kg^-1^)	8.02 ± 0.26b	8.36 ± 0.44b	8.52 ± 0.16b	8.37 ± 0.30b	9.47 ± 0.90ab	9.57 ± 1.29ab	10.74 ± 0.43a
AB(mg kg^-1^)	52.14 ± 1.11a	43.43 ± 8.59ab	33.82 ± 3.02bc	30.84 ± 1.59c	31.59 ± 0.67c	27.24 ± 2.97c	27.83 ± 1.69c
AMo(mg kg^-1^)	0.13 ± 0.00a	0.12 ± 0.00b	0.10 ± 0.00bc	0.10 ± 0.00cd	0.09 ± 0.00cde	0.08 ± 0.00de	0.08 ± 0.01e

The same letter in the same line means the difference is not significant (p<0.05).

#### 3.1.3 Effect of acidification on medium elements content

With the decrease in soil pH, the contents of calcium (Ca) and magnesium (Mg) in the soil also gradually decreased, as presented in **Table 2**; soil total calcium (TCa), total magnesium (TMg), exchange calcium (Ca^2+^) and exchange magnesium (Mg^2+^) in soil were consistent with the changes of total potassium, and the highest content at soil pH7.5 was significantly different from that at soil pH 4.5, which significantly increased by 346%, 173%, 196% and 346%, respectively (**Table 2**).

#### 3.1.4 Effect of acidification on trace elements content

Soil total iron (TFe) and available iron (AFe) content exhibited an increasing trend as soil pH decreased. The highest TFe content (12.71 g kg^-1^) was recorded at pH 4.5, while the lowest values (9.09 and 9.13 g kg^-1^) were observed at pH levels of 7.0 and 7.5 (**Table 2**). The maximum AFe concentration of 90 mg kg^-1^ and the minimum concentration of 9.09 mg kg^-1^ were measured at pH 4.5 and 7.5, both treatments were statistically significant (**Table 2**). In addition, available manganese (AMn) at pH levels of 7.0 - 7.5 was statistically significant when compared to pH 4.5 (**Table 2**).

Soil total copper (TCu) content at pH 4.5 was statistically significant when compared to pH 7.0, while the other treatments were comparable and not statistically significant (**Table 2**). The content of soil available copper (ACu) at pH 4.5 was 0.17 mg kg^-1^ higher than that at pH 7.5 (**Table 2**). The highest values of total and available zinc (TZn and AZn) content were recorded at pH 4.5, which were statistically significant compared to pH 6.0, 7.0, and 7.5. The variation trend of boron (B) and molybdenum (Mo) contents in soil is consistent with that of zinc (Zn) contents in soil, and the difference between the content of pH 4.5 and the content of pH 7.5 is very significant (**Table 2**).

In summary, the contents and availability of soil trace elementst B and Mo are the highest in the neutral range of soil pH 7. 0 and 7.5, while the contents and availability of soil Fe, Cu and Zn are the highest in the strong acidic range of pH 4.5 and 5.0.

### 3.2 Effect of simulated soil acidification on nutrient uptake of eggplant plant in greenhouse

#### 3.2.1 Effect of soil acidification on plant uptake of macro elements nutrient

**Figure 1:**
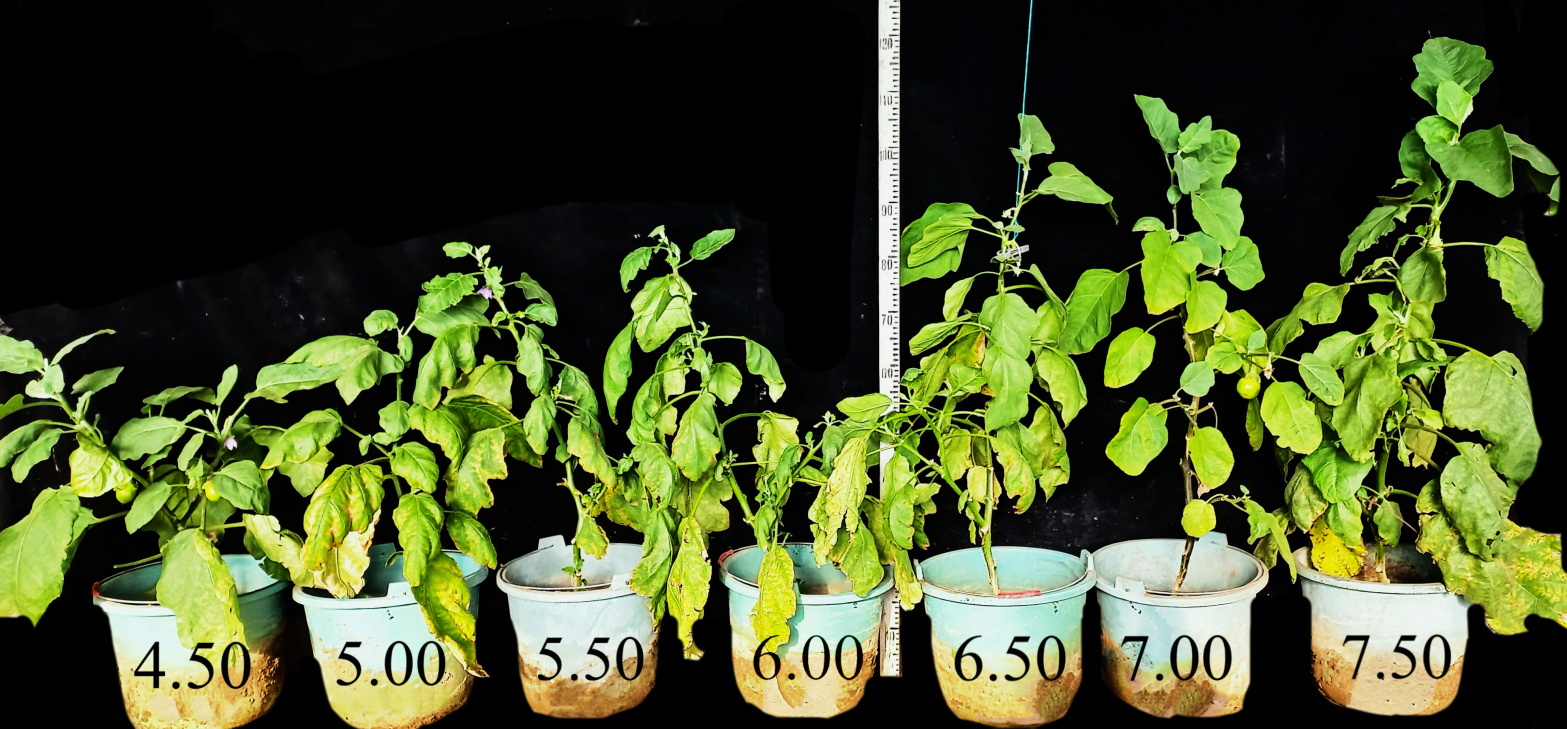
Growth performance of eggplant under varied acidification treatments.

**Figure 2:**
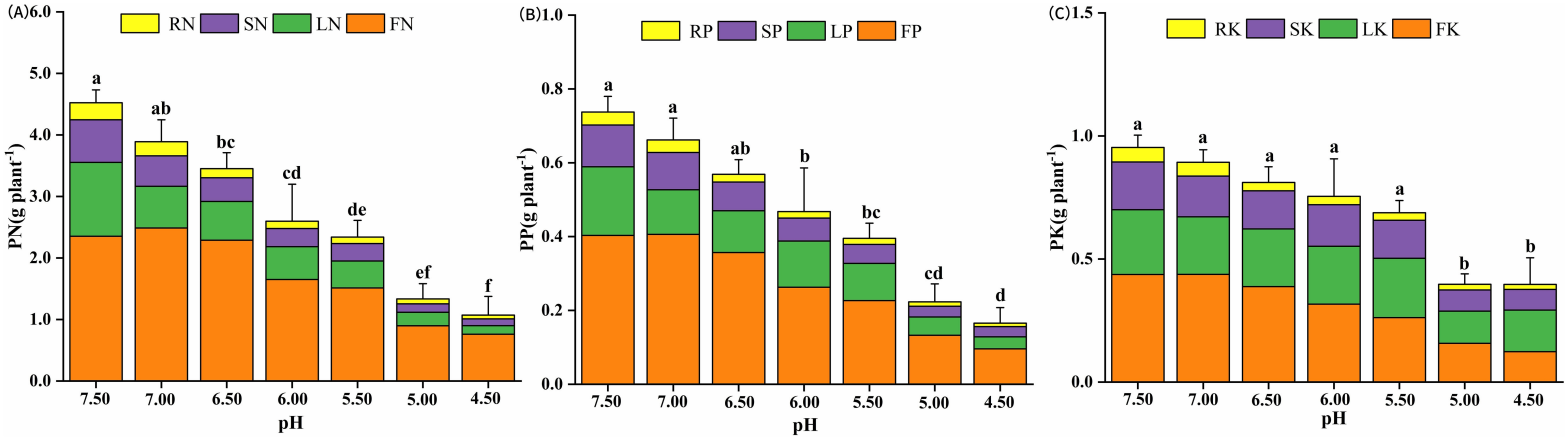
Effect of soil acidification on macro elements nutrient uptake of eggplant in greenhouse. **(A)** Total plant nitrogen; **(B)** Total plant phosphorus;**(C)** Total plant potassium (p<0.05). Different lower-case letters indicate significant differences between streatment (p<0.05).

The growth rate of eggplant exhibited a declining trend with increasing soil acidity (**Figure 1**). Meanwhile, the macro elements composition data (nitrogen, phosphorus, and potassium - NPK) in various plant parts, including root, stem, leave, and fruit tissues, were statistically significant (**Figure 2**). The total nitrogen content in plant (PN) reached its maximum at pH levels of 7.0 - 7.5, showing a significant increase of 263% - 322% compared to plant grown at pH 4.5 (**Figure 2A**). The distribution of phosphorus in each part of the plant was significant. The maximum contents of total phosphorus (PP), leaf phosphorus (LP) and stem phosphorus (SP) at pH 7.5 were significantly different from those at pH4.5 (**Figure 2B**). The absorption of fruit potassium (FK) was the highest, while the absorption of root potassium (RK) was the lowest and decreased with the decrease of soil pH. At pH 5.5 and 6.0, leaf potassium (LK) and stem potassium (SK) contents were slightly higher than pH 6.5 (**Figure 2C**).

#### 3.2.2 Effect of soil acidification on plant uptake of medium elements nutrient

**Figure 3:**
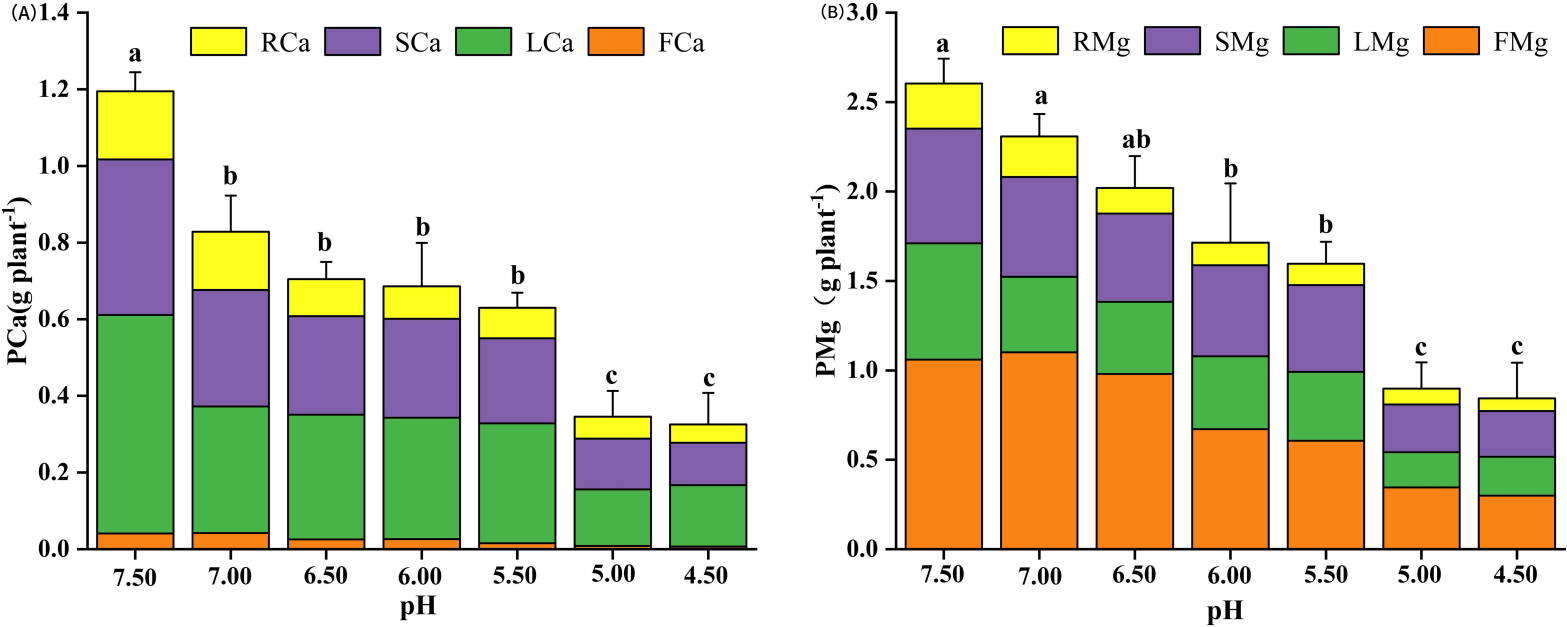
Effect of soil acidification on medium elements nutrient uptake of eggplant in greenhouse. **(A)** Total plant calcium; **(B)** Total Plant magnesium (p<0.05). Different lower-case letters indicate significant differences between streatment (p<0.05).

Soil acidification significantly reduced the distribution of calcium in plant tissues. The calcium concentration and absorption of fruits (FCa) and roots (RCa) were higher than those of leaves (LCa) and stems (SCa). The maximum total calcium content (PCa) of plant was 1.19 g plant^-1^ at pH 7.5, which was significantly different from that at soil pH4.5 (**Figure 3A**). The results showed that with the increase of soil pH, the magnesium distribution of fruit (FMg) was the highest, and that of root (RMg) was the lowest (**Figure 3B**).

#### 3.2.3 Effect of soil acidification on plant uptake of trace elements nutrient

**Figure 4:**
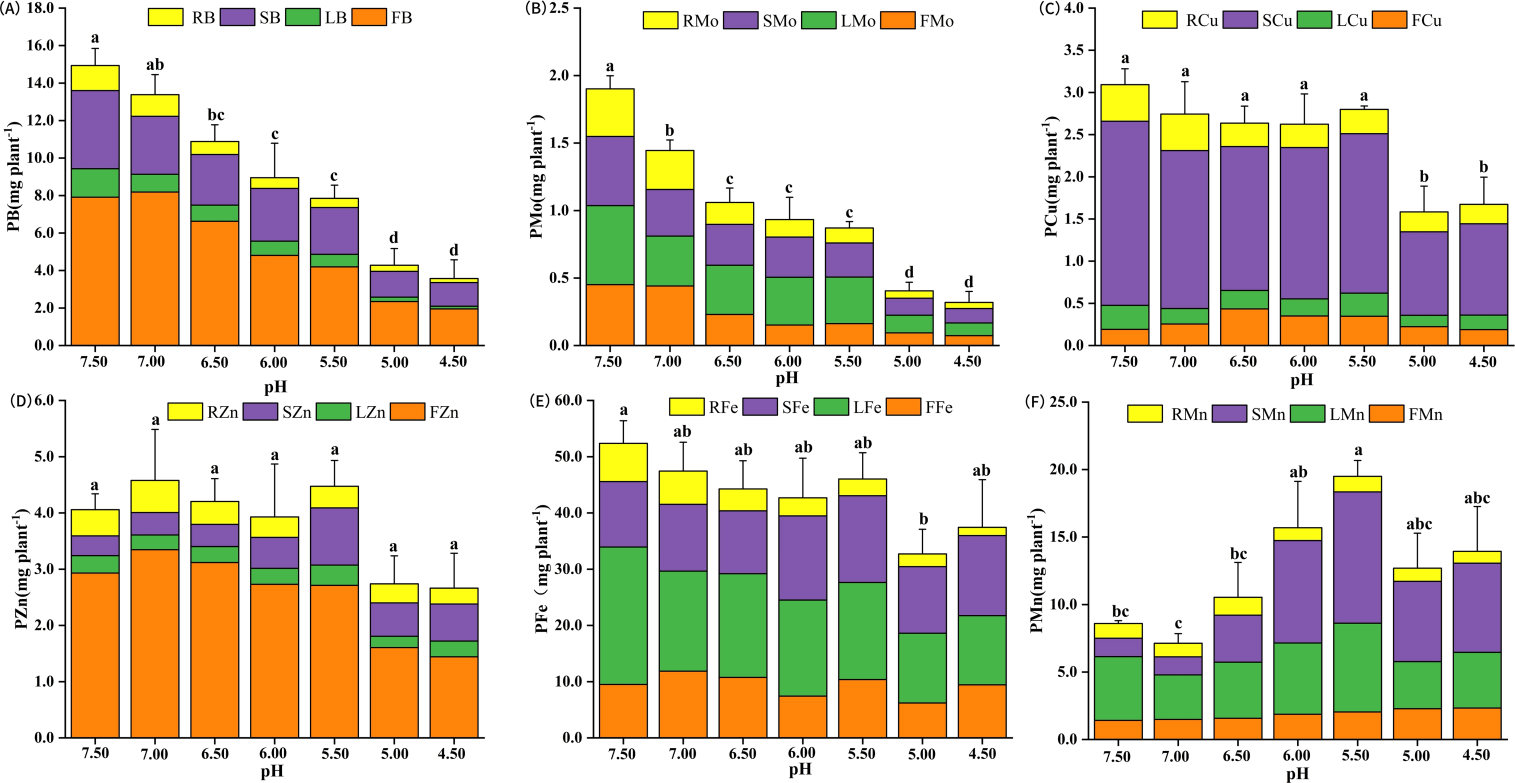
Effect of soil acidification on trace elements nutrient uptake of eggplant in greenhouse. **(A)** Total plant boron. **(B)** Total plant molybdenum. **(C)** Total plant copper. **(D)** Total plant zinc. **(E)** Total plant iron. **(F)** Total plant manganese (p<0.05). Different lower-case letters indicate significant differences between streatment (p<0.05).

Soil acidification will affect the significant absorption and distribution of trace element nutrients in eggplant plants (**Figure 4**). Most absorbed boron (B) was found in fruit tissues at pH 7.5, followed by stems. The plants total boron content (PB) at pH 7.5 was 235% - 299% higher than at pH 4.5 - 5.0 (**Figure 4A**). Furthermore, total plant molybdenum content (PMo) at pH 7.5 was significantly higher than at pH 4.5 -5.0 (**Figure 4B**). With the pH set at 6.5, the copper content in fruits (FCu) registered a significant 139% rise as compared to the value at pH 4.5 (**Figure 4C**). Total plant copper (PCu) ranged from 1.58 - 3.09 g plant^-1^, peaking at pH 7.5 and dipping at pH 5.0 (**Figure 4C**). Total plant zinc (PZn) followed similar trend, with the highest levels at pH 7.5 and the lowest at pH 4.5 (**Figure 4D**).

Data on total plant iron (PFe) content are shown in **Figure 4E**, revealing significant differences in leaf iron (LFe) and root iron (RFe) among treatments. The impact of seven pH treatments on plant manganese (PMn) content was statistically significant, with levels ranging from 7.13 - 19.49 mg plant^-1^, peaking at pH 5.0 and lowest at pH 7.0 (**Figure 4F**). In summary, the total amounts of boron (B), molybdenum (Mo), copper (Cu), zinc (Zn), and iron (Fe) were lowest at pH 4.5 and 5.0, while manganese (Mn) was highest.

### 3.3 Principal component analyses of soil acidification on nutrient uptake of eggplant in greenhouse

**Figure 5:**
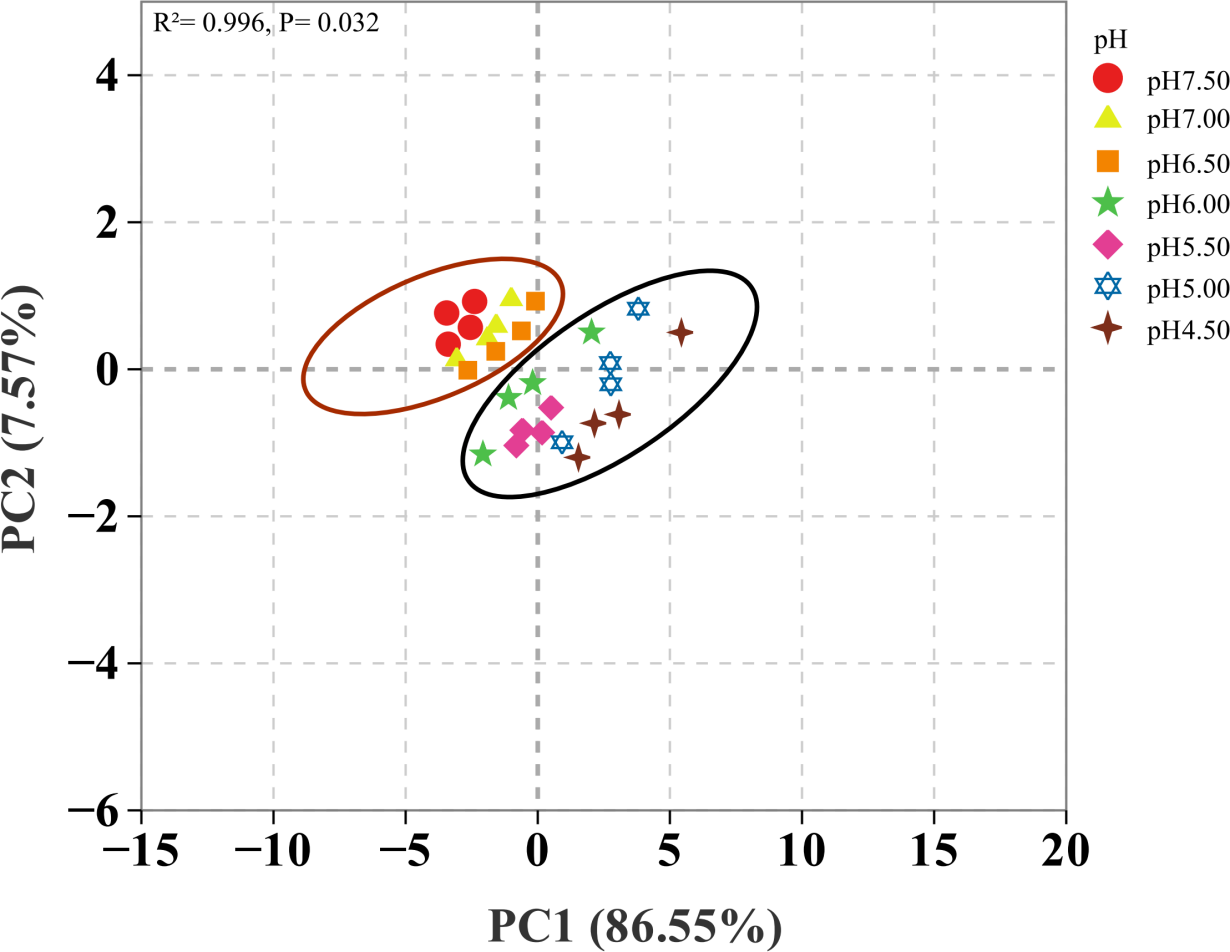
Principal component analyses of soil acidification on nutrient uptake of eggplant in greenhouse.

Principal component analyses of soil acidification on nutrient uptake of eggplant (**Figure 5**). PCA analyses is used to demonstrate the effects of different simulated acidification treatments on plant nutrients. PCA analyses shows that the nutrient distribution capacity of plant organs at soil pH 4.5, 5.0, 5.5 and 6.0 is well separated from the other four acidity ranges on the PCA1. And the two axes together explained 94% of the total variation. Adonis results also confirmed that soil acidification had a significant effect on plant nutrients (R^²^=0.996, *P*=0.032).

### 3.4 Comprehensive analyses of nutrient absorption of greenhouse eggplant by soil acidification

As can be seen from **Table 3**, the three most important chemical factors for macro elements nutrient uptake of eggplant in greenhouse under simulated soil acidification conditions were Mg^2+^ (r=0.99, *p*=0.001), pH (r=0.60, *p*=0.001) and TB (r=0. 59, *p*=0.001). The three most important chemical factors of medium elements nutrient uptake in eggplant plants in solar greenhouse were Mg^2+^ (r=0.94, *p*=0.001), TCa (r=0.61, *p*=0.001) and pH (r=0.58, *p*=0.001). The three most important chemical factors of trace elements nutrient uptake in eggplant in greenhouse were Mg^2+^(r=0.69, *p*=0.001), pH (r=0.32, *p*=0.001) and TB (r=0.30, *p*=0.001). In conclusion, soil pH and Mg^2+^ play a crucial role in regulating plant nutrient uptake under simulated acidification conditions. Therefore, we should monitor and control the soil pH in the greenhouse and avoid the application of excessive and concentrated physiological acids and nitrogen-containing fertilizers to ensure the healthy growth of eggplant in the greenhouse.

**Table 3:** Mantel test of acidification on the relationship between nutrient uptake and soil basic chemical properties of eggplant in greenhouse.

Soil properties	Macro elements in plant		Medium elements in plant		Trace elements in plant	
	Mantel r	*P*	Mantel r	*P*	Mantel r	*P*
pH	**0.60**	**0.001**	**0.58**	**0.001**	**0.32**	**0.001**
EC	0.44	0.001	0.43	0.001	0.20	0.007
Al^3+^	0.32	0.122	0.31	0.002	0.01	0.333
SOM	0.13	0.036	0.12	0.133	0.06	0.258
TP	0.31	0.019	0.36	0.014	0.32	0.059
AP	0.33	0.010	0.28	0.016	0.02	0.369
TN	0.08	0.169	0.08	0.189	0.02	0.339
AN	0.25	0.036	0.21	0.058	0.07	0.212
TK	0.28	0.027	0.25	0.052	0.26	0.002
AK	0.39	0.001	0.43	0.001	0.24	0.006
TCa	0.57	0.001	0.61	0.001	0.39	0.005
Ca^2+^	0.58	0.001	0.58	0.001	0.33	0.004
TMg	0.40	0.001	0.40	0.001	0.20	0.007
Mg^2+^	**0.99**	**0.001**	**0.94**	**0.001**	**0.69**	**0.001**
TZn	0.32	0.005	0.36	0.002	0.34	0.017
AZn	0.28	0.012	0.31	0.009	0.24	0.059
TMn	0.02	0.354	0.04	0.317	0.03	0.334
AMn	0.33	0.001	0.30	0.001	0.16	0.019
TFe	0.42	0.001	0.46	0.001	0.29	0.023
AFe	0.43	0.001	0.40	0.001	0.23	0.002
TCu	0.20	0.040	0.21	0.041	0.10	0.151
ACu	0.20	0.067	0.22	0.066	0.06	0.229
TB	0.59	0.001	0.52	0.001	0.30	0.001
AB	0.31	0.001	0.30	0.019	0.13	0.154
TMo	0.47	0.001	0.43	0.001	0.26	0.004
AMo	0.53	0.001	0.55	0.001	0.31	0.015

Bold indicates the most influential factors.

## 4 Discussion

### 4.1 Effect of simulated soil acidification on soil physicochemical properties in greenhouse

Soil nutrients are essential for the growth and development of plants. However, intensive use of chemicals in all cropping systems led to environmental pollution and soil degradation issues (Leghari et al., 2024). In greenhouse conditions fertilizers are excessively applied, which resulted in the soil acidification and consequently affected nutrient availability in eggplant (Wang et al., 2022). The continued application of dilute sulfuric acid results in a decrease in soil pH, leading to an increase in soil EC value (Zhang et al., 2020; Zhou et al., 2020). In this study, EC was observed to be highest, ranging from 1.70 - 2.01 ms cm^-1^, at pH levels between 5.00 - 4.50 (**Table 1**, *p* < 0.05). This increase in H^+^ concentration significantly impacts the alteration of physicochemical properties (Junior et al., 2020; Raza et al., 2020). Soil acidification contributes to the depletion of essential basic cations, including K^+^, Ca^2+^, and Mg^2+^ (Cai et al., 2021; Du et al., 2024b), which aligns with the findings of the present study. Meanwhile, the results concerning Mg^2+^ in our research diverge from those reported by Vogels et al. (2024). This inconsistency may be explained by the increased production of H^+^ under acidic conditions, which can lead to enhanced leaching losses of Ca^2+^ and Mg^2+^ (Cusack et al., 2016). In addition, soil acidification may diminish the soil’s capacity to retain N, P, K, and other essential nutrients (Li et al., 2018; Bai et al., 2020; Cai et al., 2021).

Soil acidification leads to the production of toxic substances, such as Al^3+^, which can accumulate significantly when soil pH< 5.00 (Wang et al., 2023b; Borges et al., 2024; Du et al., 2024a) This phenomenon restricts the availability of phosphorus in the soil (Brownrigg et al., 2022; Xie et al., 2022; Baccari and Krouma, 2023). The highest concentration of Al^3+^ recorded was 113.40 mg kg^-1^ at soil pH of 4.5 (**Table 1**), which is 5 - 6 times the critical threshold for aluminum toxicity symptoms in plant (Cai et al., 2017). Under acidic soil conditions, aluminum is released into the soil solution in its active and toxic form, reaching levels that can inhibit root growth and damage root, there by hindering the growth and development of eggplant (Bonomelli and Artacho, 2021). Some scholars have found that increasing soil acidification leads to an imbalance in soil nutrients, while heightened acidity reduces the decomposition rate of soil organic matter. In addition, a study on the excessive fertilization of tomatoes in solar greenhouses revealed that the content of AN remains at a moderate level (Lv et al., 2020). However, the finding that the content of AP was four times higher than the relative abundance standard aligns with this experiment’s results (**Table 3**, *p* < 0.05), yet it contradicts the findings of Wang et al. (2023b). This discrepancy may be attributed to the increased production of phosphatase in acidic soils (Long et al., 2016; Hafez et al., 2022; Wang et al., 2022).

In addition, it is important to highlight that as soil acidity increases, the content of soil AK tends to rise. This may be explained by the secretion of lower organic acids by the root system, which increases the net negative charge on the soil surface, there by facilitating the accumulation of K^+^ in the soil (Wang et al., 2022; Liu et al., 2023; Wang et al., 2023b). Consequently, soil pH is a critical factor in regulating various soil properties (Hong et al., 2019; Wan et al., 2020; Mosley et al., 2024).

### 4.2 Effect of simulated soil acidification on nutrient uptake of eggplant in greenhouse

The ability of crops to absorb and utilize various nutrients is significantly influenced by soil acidification. Under acidic conditions, plants encounter difficulties in absorbing and utilizing essential elements necessary for their growth and development. Our findings demonstrate that plants absorb higher concentrations of Ca and Mg, a result that aligns with the observations made by Wang et al. (2024). Mielcarek et al. (2023) reported that the P content in tomato plants decreased as pH increased, with the highest absorption of N, P, and K occurring at pH of 6.50. In our study, the absorption of N, P, and K by eggplant was highest at pH of 7.50 and lowest at pH of 4.50. These results indicate that the absorption of macronutrients by plant is severely inhibited under acidic conditions, with EC also identified as a primary factor affecting the absorption of substantial amounts of elements by plants. In addition, soil pH significantly impacts the absorption of trace elements (Yang et al., 2019). For instance, when soil pH decreased from 7.50 - 4.50, the absorption of B by plants declined from 4.19 - 2.30 g plant^-1^, and the absorption of Mo exhibited a significant decreasing trend (Shi et al., 2018; Zhang et al., 2021).

Through our analyses, we hypothesized that soil acidification significantly impacts the nutrient absorption of eggplant, potentially by altering the availability of soil nutrients. For instance, soil acidification can lead to the release of substantial amount of Al, which inhibits the uptake of essential nutrient elements such as P, K and Fe by plant roots, as confirmed by Baccari and Krouma (2023). This occurs because acidification diminishes the soil’s capacity to retain and supply fertilizers, exacerbates the loss of essential elements, and reduces the overall availability of soil nutrients. Furthermore, the quantity of nutrients absorbed by plants is contingent upon a specific subset of nutrients available in the surrounding soil (Brownrigg et al., 2022). The Mantell test revealed for the first time that the soil pH of eggplant grown in a solar greenhouse under simulated acidification significantly affects the amount of nutrients absorbed by the plants, thereby demonstrating that soil nutrient content is closely linked to the nutrient absorption capacity of the plants (Sánchez-Moreno and Curiel Yuste, 2022).

## 5 Conclusions

Elevated soil acidity significantly impacts the nutrient absorption process in eggplant. The SOM dropped to 325 5.57g kg^-1^ at pH 4.50, while EC and Al^3+^ peaked at 2.01 ms cm^-1^ and 113.40 mg kg^-1^, respectively. At this pH level, the concentrations of macroelements, medium elements, and trace elements (B and Mo) were minimized, whereas the levels of other nutrients were maximized. Elements deficiencies in soil can diminish nutrient content in eggplant, with soil pH being the main factor. The findings provides useful insights for maintaining soil health and promoting sustainable greenhouse vegetable. However, present study primarily focused on the relationship between soil nutrients and plant nutrients. The future research should navigate the dynamic association between rhizosphere microorganisms and plant nutrient uptake in the context of soil acidification. This research is crucial for maintaining soil health and promoting sustainable greenhouse vegetable production.

## Data Availability

The original contributions presented in the study are included in the article/**Supplementary Material**. Further inquiries can be directed to the corresponding authors.
