# Supplementary material for: Effect of simulated acidification on soil properties and plant nutrient uptake of eggplant in greenhouse
Source: Front Plant Sci. 2025 Mar 31;16:1558458. doi: 10.3389/fpls.2025.1558458 (PMC11994667; doi:10.3389/fpls.2025.1558458)
Supplement: Supplementary file 1 [file Table1.docx]

Supplementary Material

**Supplementary Table 1** Soil chemical properties of eggplant in greenhouse

|  | pH | EC(ms cm^-1^） | SOM(g kg^-1^) | TN(g kg^-1^) | TP(g kg^-1^) | TK(g kg^-1^) | AN(mg kg^-1^) | AP(mg kg^-1^) | AK(mg kg^-1^) |
| --- | --- | --- | --- | --- | --- | --- | --- | --- | --- |
| Soil | 7.24 | 0.28 | 19.67 | 2.31 | 1.10 | 28.23 | 38.50 | 78.74 | 721.24 |

| pH | 4.50 | 5.00 | 5.50 | 6.00 | 6.50 | 7.00 | 7.50 |
| --- | --- | --- | --- | --- | --- | --- | --- |
| Final pH | 4.45±0.05 | 5.09±0.05 | 5.50±0.09 | 5.86±0.09 | 6.62±0.08 | 7.15±0.05 | 7.47±0.15 |

**Supplementary Table 2** The final pH range for each treatment
